# From health for all to universal health coverage: Alma Ata is still relevant

**DOI:** 10.1186/s12992-018-0381-6

**Published:** 2018-07-03

**Authors:** Kiran Raj Pandey

**Affiliations:** Center for Health and the Social Sciences, University of Chicago, 5841 South Maryland Avenue, MC 1005, Suite 200, Chicago, IL 60637 USA

**Keywords:** Health for all, Universal health coverage, Health services, Health systems

## Abstract

With increasing adoption of universal health coverage (UHC), the health for all agenda is resurgent around the world. However, after a promising start the first time in 1978, the health for all agenda fizzled over the next decade. This commentary discusses the origin of the health for all agenda in the 1970s and the influence of global politico-economic forces in shaping that agenda, its demise and the resurgence in the form of UHC in the twenty-first century. We discuss UHC’s focus on finances and the increasing role of market economy in health care, and the opportunities and risks UHC poses. We conclude by saying that UHC’s greater focus on finances is prudent, but in order to achieve its promise, UHC needs to regulate the market based provision of healthcare, and incorporate more of the people and community centered ethos of its earlier iteration from 40 years ago.

## Background

Health for all, under the rubric of universal health coverage (UHC), is a resurgent rallying cry in global health. In December 2017, at the universal health coverage forum in Tokyo, governmental and multilateral organizations signed the Tokyo declaration re-pledging their allegiance to UHC agenda, and December 12 is now marked as universal health coverage day [1]. However, this is not the first time health for all has been the flagship global health policy agenda. 40 years ago in the 1970s, under the leadership of Halfdan Mahler, the World Health Organization (WHO) championed a health for all agenda, culminating in the ground breaking primary health care conference in Alma Ata (today Almaty), Kazakhstan [2]. The Alma Ata declaration of health for all by 2000 was signed with overwhelming support of governments from around the world. Within a decade or so however, the momentum had largely fizzled [3].

It took almost two decades for the health for all movement to regain it’s footing. In 2005, the 58th World Health Assembly (WHA), the WHO’s governing body, adopted resolution 58.33 urging member states to reform their health financing mechanisms in order to achieve universal coverage [4]. Following this, the WHO dedicated the World Health Reports 2010 and 2013 to universal health coverage, and the 67th United Nations General Assembly in 2013 passed a resolution endorsing universal health coverage [5]. Since then, UHC has made steady purchase the world over; governments and multilateral institutions such as the WHO have adopted it as their major health policy plank, and UHC is now even part of the sustainable development goals. But how does the UHC agenda differ from the earlier health for all agenda? In this commentary, we look at the origins of health for all and UHC vis-a-vis the global political economy of the last 40 years, the potential opportunities and risks of UHC, and what UHC can learn from its earlier iteration.

## Main text

A useful point to start a comparative analysis of the health for all and the universal health coverage agenda is to look at the documents that underpin the tenets of these two ambitious health policy initiatives: the Alma Ata declaration and WHA resolution 58.33. The 1978 Alma Ata declaration focused on primary health care as a means to achieving health for all, with a focus on community participation; community needs and priorities, their values and their vision for a health care system were seen as the guiding principles of achieving health for all [2]. In that sense it was an idealistic vision of a community led health system centered around the wellbeing of people. By comparison, in the WHA resolution 58.33, there is fleeting mention of the nature of health care systems or services that will be supported, except that a basket of essential services will be covered without elaborating what those essential services might be. The major focus of UHC has steadily been that people will not have to suffer from financial hardship while accessing health care. The World Health Report 2010 on UHC was dedicated to health systems financing. The Alma Ata declaration mentioned financial considerations in passing.

This difference is important. Alma Ata’s ignoring finances would become its greatest folly. Giving financial considerations a short shrift drew partly from the fact that the Alma Ata conference and the declaration came with less preparation than their proponents would have liked. But it also drew from the fact that the Alma Ata declaration was an idealistic vision much like the declaration of New International Economic Order (NIEO) which formed the moral and political backdrop for the health for all movement [6]. The United Nations led 1974 declaration of NIEO aimed to tilt the global economic order in favor of the developing economies of the global south, but it failed to elucidate a pragmatic pathway on how that could be achieved, except to imply that it was incumbent upon developed economies to renege on their economic interests in favor of developing countries for the cause of global social justice. That idealism failed at the cross winds of global political economy, and so would the similarly idealistic health for all movement.

By the early 1980s, a wave of neoliberalism had swept both sides of the Atlantic. The rich world used the Bretton Woods institutions—the International Monetary Fund (IMF) and the World Bank—to impose austerity on cash strapped developing economies by tying loan terms to a series of policies meant to expand the market economy and shrink public spending, mostly in social sectors like health. With these policies in place, the vision for health for all died a predictable death [7]. Over the next two decades, publicly owned health systems in many aid dependent developing countries were systematically underfunded and user fees were introduced, reducing access to health services for people who needed them the most. In their place, piecemeal health interventions like immunization and growth monitoring were introduced through out much of the developing world, under what was labeled selective primary health care [8]. Although there were improvements in health in selective areas like child health as a result of this, the inadequacies of the global health systems were soon laid to bare by the AIDS epidemic, and the flares of several other health emergencies such as Influenza, Cholera and Ebola epidemics. In the absence of a functioning health system, mounting up a response against these epidemics was hard, and prohibitively expensive [9, 10].

Seen against this reality, a resurgent health for all agenda premised on UHC not only offers a chance to push back at the damage done on public health systems in the past few decades, but also the possibility of expanding health care to everyone who needs it. However, challenges remain. The last 25 years of discourse in global health, starting with the publication of the World Development Report 1993, has mainstreamed the role of the marketplace in health around the world [11]. The UHC agenda operates out of acceptance of that role. Economic utility drives the rationale for UHC. The overarching discussion of current UHC agenda is to make sure that people can pay for health care, some or a lot of which may be provisioned at the marketplace. What hasn’t been as forcefully discussed thus far is: what kind of health care? And to what end?

That the market economy tends to fail when it comes to health care has been noted before [12]; pooled funds can be used to ameliorate or exacerbate these failures. Already, examples of detachment between market based provision of health care and people’s health care needs are starting to emerge. Starting with the market expanding policies of the eighties and nineties, when health care started opening up to the private sector in the developing world, several south Asian countries saw a flood of poorly regulated private hospitals and medical schools open up. These expanded access to health care and medical education to some extent, but poorly regulated for profit medical schools often trained the highest paying students, mostly poorly, in procedure oriented fields that barely match community needs [13, 14]. Private hospitals in the developing world today often hold clinician-management meetings to increase consumption of revenue boosting imaging and diagnostics (author’s personal observation) and proffer “executive health packages” of questionable value [15]. Countries like China and Brazil have seen an alarming increase in the number of unwarranted cesarean section, a large portion of which correlates with the increase in the number of private hospitals and the provision of health insurance [16–18]. Private insurance pools in India have reported hospitalization rates that are 2–3 times the normal, often with no additional benefit [19].

A market economy in health care brings in new investment and ideas and offers people an alternative—as a counter example to the rampant dereliction and neglect public health systems suffer in most of the developing world [20]. However, unchecked and unregulated, it runs the risk of reducing health care down to a freely tradable industrial good or service [21]. There appears to be an increasingly deeper wedge between the intervention oriented ethos of private health care systems and the needs of communities that these systems aim to serve. In many low and middle income countries with public and private provision of healthcare, the introduction of insurance has had the net effect of funneling resources from publicly provisioned primary care to privately provisioned intervention oriented secondary or tertiary care [22]. Overly relying the delivery of health care on the consumption of discrete health interventions with the aide of technology laden processes provided by a professional class of providers runs the risk of removing the “care” element out of our health care systems [23]. UHC can not facilitate such industrialization of health care. Additionally, consumption of health services alone is unlikely to improve health outcomes; even in the developing world where lack of access to care has been assumed as the reason for poor health outcomes, merely increasing access to care has failed to adequately improve health outcomes [24].

Ensuring UHC is an inherently contentious and vexing political process, and the process can easily tilt against UHC when a small group of people motivated by a reckless profit motive abuse the system to the detriment of the larger majority [25]. This can put UHC in a tailspin even before it has an opportunity to come to fruition. Furthermore, while de jure declaration of universal health coverage can be popular, countries and communities that have made significant health gains have done so not only by merely increasing access to health services, but by dint of the hard work of simultaneously addressing the more distal causes of ill-health [26, 27]. Edwin Chadwick, who authored a landmark report on sanitation, called health promotion more of a civil engineering problem than a medical problem, hinting at the cause of majority of the ill health in the nineteenth century [28]. A variation of that basic observation is true even today. However in order to address these issues, UHC needs to expand the debate further afield from just ensuring financial protection and access to care, to a renewed focus on overall health and wellbeing.

A visit back to the ideals and tenets of Alma Ata could help rectify these issues with UHC. People, their communities and their wellbeing need to be back at the center of discussion on UHC. Values of economic utility may have made UHC more practicable, but adopting humanistic values of solidarity and obligation could help it iron out the drawbacks outlined above [29]. UHC should enable health services and systems to develop into a public commons, as opposed to a commodity, that can achieve the greatest common good for the most people [30, 31]. Although there seems to be increasing recognition of the need to make UHC more people centered, as spelled out in the WHO’s Integrated People Centered Health Systems framework, this recognition has yet to see mainstream operationalization [32]. A lot of that operationalization requires structural and organizational reconfiguration of our healthcare systems. That might mean taking the health system much closer to the community, even to the point of de-professionalization of certain aspects of the care function. Selective de-professionalization of the care function, as has been done with community health and health extension workers for example, enables people and communities to become producers of health care as envisioned by Alma Ata, as opposed to mere consumers of services produced by a professional class of providers in corporate institutions detached from the community [33]. The role of the family and community in health care production needs to expand. Community production of health care helps narrow the ever increasing wedge between communities and their health systems. It also has the potential to make health care more humane, self-reliant, and less financially burdensome [34]. Furthermore, with an epidemiological shift from acute to chronic illnesses that require long term, low intensity, continual care, alongside increasing economic and social progress, greater ease of information access, and a move toward empowerment of people and communities, family and community production of health care is an imperative as well as a distinct possibility [35].

Delegating the locus of strategizing and decision making process from multilateral and donor institutions to national and provincial governments, and delegating implementation to the local governments and communities is an important way of ensuring the acceptability and viability of UHC, another idea espoused by Alma Ata that is only barely beginning to find recognition with UHC [1]. Local governments and communities are more likely to not only know what the local problems are but also what the local communities value. Given that people and communities may differ on how they accept disease and death, it is also hard to argue that every community would choose to spend ever increasing sums of money on consuming more health services in the hopes of marginally extending life, especially if the financial trade-off involves compromising on livelihood, education and pensions [36]. Community oriented health systems are also likely to better tackle the distal causes of ill health in their communities, while reducing the need for health services and costs in the first place [37, 38].

## Conclusion

The current UHC agenda, offers great promise in mobilizing funding for health systems around the world, but with a great focus on increasing resources available for health care and alleviating financial burden without a concurrent vigorous discussion on what care is provided and how, UHC runs the risk of being held hostage to the machinations of a publicly buttressed but poorly regulated health services market economy, which is to value profit over health. In such a scenario, universal health coverage may be able to increase access to health services but the trade offs that are involved may make UHC unpopular and unsustainable. That would lay to waste a great opportunity. Already some developed nations with runaway health care costs are starting to feel the pinch. The developing economies of the world that have a much smaller fiscal space to play with, will feel this pinch much before they can even extend health coverage to everyone. UHC’s focus on finances is prudent, but revisiting the people and community centered ethos of Alma Ata might help UHC overcome the challenges it faces and achieve its true promise.

## Abbreviations

AIDS: Acquired Immune Deficiency Syndrome
IMF: International Monetary Fund
NIEO: New International Economic Order
UHC: Universal Health Coverage
WHA: World Health Assembly
